# Essential Oils as Potential Alternative Biocontrol Products against Plant Pathogens and Weeds: A Review

**DOI:** 10.3390/foods9030365

**Published:** 2020-03-21

**Authors:** Robin Raveau, Joël Fontaine, Anissa Lounès-Hadj Sahraoui

**Affiliations:** Unité de Chimie Environnementale et Interactions sur le Vivant (UCEIV, UR 4492), Université du Littoral Côte d’Opale, SFR Condorcet FR CNRS 3417, 50 rue Ferdinand Buisson, 62228 Calais cedex, France; raveau@univ-littoral.fr (R.R.); fontaine@univ-littoral.fr (J.F.)

**Keywords:** essential oils, biological properties, crop protection, sustainable agriculture

## Abstract

Naturally produced by aromatic plants, essential oils (EO) contain a wide range of volatile molecules, including mostly secondary metabolites, which possess several biological activities. Essential oils properties such as antioxidant, antimicrobial and anti-inflammatory activities are known for a long time and hence widely used in traditional medicines, cosmetics and food industries. However, despite their effects against many phytopathogenic fungi, oomycetes and bacteria as well as weeds, their use in agriculture remains surprisingly scarce. The purpose of the present review is to gather and discuss up-to-date biological activities of EO against weeds, plant pathogenic fungi, oomycetes and bacteria, reported in the scientific literature. Innovative methods, potentially valuable to improve the efficiency and reliability of EO, have been investigated. In particular, their use towards a more sustainable agriculture has been discussed, aiming at encouraging the use of alternative products to substitute synthetic pesticides to control weeds and plant diseases, without significantly affecting crop yields. An overview of the market and the recent advances on the regulation of these products as well as future challenges to promote their development and wider use in disease management programs is described. Because of several recent reviews on EO insecticidal properties, this topic is not covered in the present review.

## 1. Introduction

Plants are naturally able to produce a wide range of molecules, especially secondary metabolites, which are known to perform a function in protecting plants against pathogens, owing to their biological properties [1]. Among these molecules, more than 3000 essential oils (EO), which are complex mixtures mostly constituted of secondary metabolites, are identified and known [2]. Many of the EO are known for centuries for their anti-septic, antioxidant and anaesthetic properties and a lot among them have been reported for their use in traditional medicine. Essential oils constitute an important source of biologically active compounds—antibacterial, insecticidal, fungicidal, nematicidal, herbicidal, antioxidant and anti-inflammatory [2,3,4,5]. Three hundred of them are commercialised and frequently used in cosmetics and flavours as well as in the food industries [2,6]. They are also used in the food sector as spices or to prepare beverages [7].

Biological control is not a new concept and it is gaining a lot of interest recently, for the integrated management of crop pests. Biocontrol products are classified into four main classes, including macro-organisms, microorganisms, semiochemical products and natural substances originating from plant, algae, microorganisms, animal or mineral sources [8]. Essential oils are found amongst all natural substances of vegetable origin and therefore considered as potential biocontrol products. The current overuse of synthetic pesticides, causing environment and human health negative effects and pesticide-resistant biotypes, the emergence of resistance phenomena and the pesticides’ withdrawal and restrictions (Directive 91/414/EEC, July 1993 and Regulation 1107/2009/EC, 2011) on a European but also on a worldwide scale, are encouraging a reduction in pesticides’ use and the need for alternative control methods and integrated pest management (IPM) systems [9].

With a better acknowledgement of IPM approaches [10], biocontrol products and especially EO have a significant interest as they are bio-sourced products regarded as more ecological and alternative solutions in comparison with synthetic pesticides which show greater environmental and human health risks [11,12]. In that matter, the use of biocontrol agents and EO as substitutive solutions to synthetic pesticides is greatly encouraged in Europe by the directive 2009/128/CE, which aims to reduce the application of pesticides and promote the introduction of molecules and agricultural inputs more in line with sustainable development (http://data.europa.eu/eli/dir/2009/128/oj). Nonetheless, parameters influencing EO activity or efficiency need to be investigated, in order to legitimate their use as alternative methods to pesticides, alone or in addition to other molecules.

Thus, the current review focuses on gathering information and results from various studies on EO properties—with a focus on biological activities against weeds and plant pathogens, affecting crops pre- or post-harvest, in particular fungi, oomycetes and bacteria. An overview of the market and the recent advances on the regulation of these products as well as future challenges to promote their development and wider use in disease management programs is described. As insecticide use has been the most reviewed biological property, in comparison with other ones against plant pathogens, with the recent contribution of several authors on that subject [13,14,15,16,17,18,19], as well as in several book chapters [20,21,22], it is not covered in the current paper.

### 1.1. Specificities of Essential Oils

Naturally produced by aromatic plants and commonly obtained by hydrodistillation or steam distillation, EO are synthetized by all aromatic plant organs, flowers, buds, leaves, seeds, fruits, roots and rhizomes, wood and bark in relatively small amounts. They are located and stored in secretory cells, cavities or canals, epidermic cells or glandular hairs [3,4].

Either colourless or with a colour ranging from pale yellow to brown, these oils are commonly liquid at room temperature, but densities may be very different, and some oils can be resinous or even solid [23]. Poorly soluble in water but highly soluble in organic solvents, they are classified as fat-soluble [24].

Essential oils are usually rich in various compounds, comprising 20 to 60 active substances, and in many cases, can be characterized by up to three major components, at a relatively high concentration compared to other compounds present in trace amounts [2,3]. For example, linalool (68%) is found in *Coriander sativum* EO, limonene (54%) and α and β-pinene (respectively 7 and 3.5%) in *Pinus pinea* EO, carvacrol (65%) and thymol (15%) in *Origanum heracleoticum* EO and menthol (59%) and menthone (19%) are found in *Mentha x piperita* EO [3,25,26]. The major components found in EO are often responsible for their biological properties and can be gathered in two main groups:

- Terpene hydrocarbons, constituted of monoterpenes and sesquiterpenes. Monoterpenes represent 80% of the EO’s composition [27,28].
- Oxygenated compounds, constituted mostly of alcohols, phenols, aldehydes and esters. The aromatic and oxygenated compounds occur less in EO than terpenes but are yet frequent [3,29].

The chemical composition of the EO varies, depending on the organ the EO is extracted from [29,30,31]. As an example, EO from *Salvia officinalis* displayed a significantly different composition, whether it was distilled from leaves, stems or flowers. In fact, α-thujone was the major identified compound, respectively representing 30, 55 and 18% of the EO compositions. Similarly, camphor which was identified in the EO distilled from the three different organs, varied from 19.5 to 3.5% (respectively in the EO from leaves and flowers [31]). In addition, for a same plant species, EO’s yield and chemical composition are wildly variable under the influence of several parameters, depending on growth and development conditions of the plant they originate from, climatic conditions (temperature, rainfall, humidity, light intensity), culture site (soil composition, acidity, pollution and mineral nutrition availability), harvesting time [30,31,32] and the root colonisation by symbiotic microorganisms, in particular arbuscular mycorrhizal fungi [33,34]. Differences in terms of chemical composition also appear between plant species of the same genus and more precisely between varieties of the same plant species, especially regarding the main compounds’ proportions [35,36].

Owing mostly to their volatile nature and to the thermolability of their components, EO are very susceptible to degradation [5,37]. First, because of the close structural relationship between molecules, they may easily convert into each other through different processes, triggered by various factors which may affect them during storage or use, causing their degradation [5,38]. This occasional degradation is possible to assess through several chemical indexes (peroxide index, acid index, etc.,), physical measurements (refraction index, density, ethanol miscibility, etc.) or chromatographic analyses [5,37].

Among all the degradation ways known, oxidation, isomerisation, polymerisation and dehydrogenation are the most frequent ones [5]. In practical terms, EO’s degradation is affected by several chemical and environmental factors, influencing first the likelihood of EO to be altered and then the reaction’s process. External factors including temperature, light and oxygen availability and the presence of impurities in EO as well as the nature of EO compounds and their structure may be determinant regarding EO’s stability [5].

Chemical molecules are most of the time very susceptible to temperature variations. In lemon EO an increased temperature leads to a drop in geranial, neral and β-phellandrene concentrations, whereas an increase in *p*-cymene, limonene oxide and geranic acid amounts [39]. Besides volatilization, oxidation reactions may occur under thermic stresses. These reactions are divided into different categories: oxidative cleavage of carbon-carbon double bonds, dehydrogenation leading to aromatic cycle formation, epoxide formation and allylic oxidation resulting in alcohols, ketones and aldehydes apparition [40]. As an example, terpenoids are known to be both volatile and heat sensitive and may either be easily oxidized or hydrolysed, based on their structure [5].

Essential oils are also very sensitive to light radiation. More specifically, it has been shown that changes in EO composition occurred in light (in comparison with a storage in dark conditions), especially an oxidation of major compounds such as monoterpenes in EO from laurel and fennel. Oxidation occurs even in the dark, but at a relatively slower rate [41].

Isomerisation process is favoured by light radiations on EO as well. A modification in the composition of anise, clove or cinnamon EO, with the transformation of *trans-*anethole into *cis-*anethole as a striking feature, results in a highly increased toxicity and an unpleasant smell [42]. It is notable that for the same concentration, two aromatic molecules may have very different properties, especially olfactory ones (depending on volatility and molecular structure); if the perception threshold of the altered molecule is consequently lower for an organism, compared to the unaffected one, this might be sufficient to deteriorate the product and its efficiency [38,42].

The impact of light and temperature in presence of atmospheric oxygen has been investigated [28]. Even at low temperature, it has been shown that EO oxidation could occur and result in the formation of peroxide radicals and hydroperoxides. In fact, oxygen solubility in the EO increases with a decreased temperature (Henry’s Law). For example, in rosemary, pine, lavender and thyme EO, higher amounts of peroxides were detected at low temperature [28].

According to the previous observations, it appears necessary to find optimal conditions for EO storage. Processing EO with a non-reactive gas has been investigated, but optimal storage conditions remain unclear and only a few EO or volatiles have been subject to storage experiments so far. Nonetheless, a storage at room temperature, in the absence of both oxygen and light are highly recommended [5,37]. In addition to the three external factors presented so far, EO are also susceptible to react with the packaging material or with impurities present in the EO’s mix. Humidity rate and some metal contaminations may result in oxidation reactions, with the prior presence of hydroperoxides in the EO [43].

One should keep in mind that because of their potential degradation, EO properties may be severely affected [44]. There are numerous examples of flavouring agents losing their organoleptic properties and going through viscosity change, because of the alteration of the EO’s main compounds [5].

To summarise, a specific molecule may be affected in many ways and get altered through several degradation processes, which may eventually result in the apparition of various degradation compounds. To illustrate that observation, degradation of lemon EO at 40 °C, in presence of oxygen and copper oxide can lead to the apparition of the following compounds [39]: *p*-cymene, limonene oxide, α-terpineol and geranic acid. It is important to mention that attention should be paid to storage conditions so as to avoid unwanted degradation, that may alter the biological properties of the EO as well as exerting a potent toxicity due to the presence of alteration compounds.

### 1.2. Essential Oil’s Use in Agriculture, against Plant Pathogens and Weeds

An increasing number of EO has shown an interesting activity from an agricultural consideration, against a broad spectrum of micro-organisms *in vitro* and *in planta* and against weeds and bioindicator plants.

#### 1.2.1. Antifungal and Anti-Oomycete Properties

Phytopathogenic fungi are responsible for nearly 30% of all crop diseases [45,46] and may have a high impact on crops, affecting them during cultivation or post-harvest, during storage. From an economic concern, they can cause high yield losses by damaging host plant, whereas on a sanitary aspect, some of the fungi (*Aspergillus* sp., *Fusarium* sp., etc.,) are known to produce mycotoxins, responsible for pneumopathies or containing carcinogenic compounds. Previous studies reported EO activities against plant pathogenic fungi and major phytopathogenic fungi from the previous decade until 2010 [47,48,49]. The present manuscript focuses on the most recent contributions to the field. In fact, effects of a consequent number of different EO have been investigated toward a wide range of phytopathogenic fungi and oomycetes in the past decade (Table 1). The complexity in comparing the different results resides in the different methods used for the fungicidal assays, with their results expressed in different ways with either *in vitro* or *in planta* assessments (IC_50_, MIC and MFC—respectively half maximal inhibitory concentration, minimal inhibitory concentration and minimum fungicidal concentration—inhibition zone, etc.,). Among all the phytopathogenic fungi targeted by the described works, *Alternaria*, *Botrytis*, *Fusarium*, *Penicillium* and *Rhizoctonia* are the most studied ones (Table 1). It has been demonstrated through many studies that the response of a specific phytopathogenic fungus in contact with EO was highly variable from one EO to another: *Botrytis cinerea* is inhibited by EO from black caraway and fennel, but not from peppermint [50]. One should note that the presence of either phenolic (fennel EO) or aromatic compounds (black carraway) seem to exert a higher antifungal activity against *B. cinerea*. Similarly, *Aspergillus* sp. was shown susceptible to EO from lemongrass, clove, oregano and thyme but not susceptible to cinnamon and ginger EO [51] and *Penicillium digitatum* highly affected by thyme and summer savory EO, less by fennel and sweet basil ones [52].

The same kind of pattern has been observed with EO. *Mentha x piperita* has been demonstrated efficient against *Rhizoctonia solani* and *Macrophomina phaseolina,* showing a lower MIC than *Bunium persicum* and *Thymus vulgaris* EO [53], but less efficient in the management of *Fusarium oxysporum* [54] and *Penicillium verrucosum* [55], nevertheless expressing an antifungal activity. Furthermore, EO from Lemongrass *(Cymbopogon citratus*) was demonstrated efficient against *Colletotrichum gloeosporioides* [56] and *Aspergillus spp*. [51], exerting a high antifungal activity, but less efficient against *F. oxysporum* requiring relatively high inhibitory concentrations [57].

In addition, the results from these studies indicate that EO have the potential to target fungi affecting plants both during the cultivation or causing diseases occurring during the storage (post-harvest diseases), including in particular several species from *Penicilium* genus (*P. digitatum*, *P. expansum*, *P. italicum*), *Geotrichum citri-aurantii* or *Rhizopus stolonifer*.

#### 1.2.2. Bactericidal Properties

Bacteria causing diseases on plants may have a considerable economic impact. As an example, bacterial diseases caused by *Xanthomonas spp.* affect a wide range of host plants, causing considerable damages on plants and hence a loss in terms of yield and crop quality [84,114,115].

Over the past 5 years, a growing number of studies has been published regarding EO antibacterial properties, especially against plant pathogens, depicting a growing interest in biocontrol methods. The number of studies reporting EO as antibacterial agents in a plant pathogen perspective is still limited (Table 2), while most of the studies on antibacterial properties of EO are focusing on a food preservation or health issue perspective. 

The response and susceptibility of pathogens to EO or EO major compounds are diverse. It has for instance been demonstrated that the effect of basil EO on different bacteria induced various responses in terms of inhibition [116], against a wide range of pathogens. It has been shown particularly efficient against *Pseudomonas tolaasii*, whereas *Brenneria nigrifluens* was barely affected by the EO. Additionally, *Xanthomonas citri* and *Rhodococcus fascians* were also inhibited but at higher EO concentrations in comparison with *P. tolaasii*. Another study has shown a mitigated success of the *Tanacetum* species EO, being ineffective against *Erwinia amylovora* or *Xanthomonas* sp. [117]. *Origanum onites* has on the contrary proven itself efficient against *Clavibacter michiganensis* and *Xanthomonas* spp. with consistent inhibition zones [118].

#### 1.2.3. Herbicidal Properties

Essential oils have been investigated for their suspected effect on seed germination and shoot growth and development (inhibiting either one or both processes [130,131,132]) and are likely to be used in weeds’ control (Table 3). Several authors previously synthesized EO herbicidal and phytotoxic properties in reviews [133,134]. These phytotoxic effects have been demonstrated for *Amaranthus retroflexus, Chenopodium album* and *Rumex crispus* being completely inhibited in contact with *Origanum acutidens* EO [135], for *Raphanus sativus*, *Lactuca sativa* and *Lepidium sativum* tested with EO of *Thymus vulgaris, Verbena officinalis* and *Melissa officinalis* [136] and for *A. retroflexus, Cirsium arvense* and *Lactuca serriola* treated with *Achillea gypsicola* and *Achillea biebersteinii* EO [137]. Furthermore, visible damage on a grown weed are reported on *Parthenium hysterophorus* [138] and on little seed canary grass [139] in contact with *Eucalyptus citriodora* EO. However, a few EO have been demonstrated ineffective for the purpose of weed control. *Ocimum basilicum, Foeniculum vulgare* and *Pimpinella anisum* EO against *Raphanus sativus, L. sativa* and *L. sativum* germination and radicle growth [136] while *Achillea gypsicola* EO was shown ineffective against the germination of *Chenopodium album* and *Rumex crispus* [137].

#### 1.2.4. Essential Oil’s Mechanisms of Action

Even if *in vitro* EO biological properties against a wide range of organisms have been well covered, as mentioned previously, their mechanisms of action have scarcely been investigated. In particular, with the limited number of studies on antibacterial properties, the insights towards the understanding of the EO’s antibacterial mechanism remains very limited. Yet, a number of notable features are put forth and will be discussed in the present section.

Two recent reviews on secondary metabolites’ mechanisms of action, including EO and plant extracts (obtained through organic solvent extraction), pointed out six different mechanisms regarding antifungal properties [151,152]:

- Inhibiting the fungi cell wall formation;
- Disrupting the cell membrane by inhibiting ergosterol synthesis;
- Affecting the fungal mitochondria by inhibiting the mitochondrial electron transport;
- Inhibiting cell division;
- Interfering with either RNA or DNA synthesis and/or inhibiting protein synthesis;
- Inhibiting efflux pumps.

From the current state of knowledge, it appears that the common property of many EO is that they affect membrane permeability or functioning, leading to cell death in fungi [153] or bacteria [154]. For example, coriander EO (*C. Sativum*) has shown an activity against *Candida albicans* by binding itself on membrane ergosterol and this way increasing membrane permeability [155,156,157]. A prominent activity of terpenoid compounds present in EO has been discussed, highlighting their potential to attack and disrupt cell walls (by inhibiting β-glucans and chitin synthesis, compromising its integrity and causing the cell to lose control over its shape and disrupting homeostasis, eventually leading to cell death [151,158]) and membranes, affecting not only their permeability (and causing cell leakage) but also compromising membrane functions, such as electron transport, protein and enzyme activity or nutrient transport and uptake [84,122,159]. A similar effect has been demonstrated for *Mentha spicata* EO against *A. flavus* [66]. The inhibition of the biofilm formation has also been put forth as a key feature in EO antimicrobial mechanisms, against both bacteria and fungi [152]. In particular, numerous studies reported EO as *C. albicans* biofilm inhibitors [152]. Several EO from *C. sativum* or *Ocimum americanum* demonstrated an inhibitory effect towards *C. albicans* biofilms [152,160,161]. *Citrus* EO were also demonstrated capable of inhibiting bacterial (*P. aeruginosa)* and fungal (*A. fumigatus* and *Scedosporium apiospermum*) biofilm establishment [162]. Bound to this aspect, ‘quorum sensing’, that is the ability to detect and to respond to cell population density by gene regulation, has been demonstrated to be affected by EO [152]. As an example, *Citrus* EO previously mentioned were shown able to inhibit quorum sensing in both *P. aeruginosa* and *C. albicans,* leading to a membrane permeabilization of both organisms [163].

A study on tea tree EO demonstrated that the EO action against *B. cinerea* resulted in membrane cell permeability modifications and in a loss of cellular organelles’ function [90]. Carvacrol, found as a major compound in thyme (45%) and oregano (60 to 74%) EO, is known for its antibacterial activity against a broad range of Gram-positive and Gram-negative bacteria. It has been shown that carvacrol affects Gram-positive bacteria’s membranes and modifies their permeability regarding H^+^ and K^+^ cations. It also alters the outer membrane of Gram-negative bacteria, hence increasing cytoplasmic membrane’s permeability to ATP and unleashing lipopolysaccharides [164]. In fact, Gram-negative bacteria are suspected to be less sensitive to EO than Gram-positive ones, as they possess an outer membrane rich in lipopolysaccharides, which indeed restricts the direct contact between the EO and the cytoplasmic membrane, in contrast with Gram-positive bacteria [35,154]. Yet, the number of studies targeting Gram-negative bacteria are predominant (Table 2).

More specifically, on a molecular level, it has been shown that phenolic and terpenoid compounds were more efficient in comparison with esters, alcohols, aldehydes, etc., [152,165,166]. The presence of a phenolic core is suspected to be the reason of the greater efficiency, and that because of a hydroxyl group resulting in both antifungal and antibacterial activities [167]. The antifungal activity is also likely to be related to the steric hindrance of specific groups: more hydrophobic molecules such as phenolic compounds or aromatic aldehydes are more susceptible to exert a higher antifungal activity [167,168]. The fat-soluble property of EO has been shown essential in their antifungal activity too [169]. Hydrophobicity of EO and their components allows EO to break through lipids of cell membranes and mitochondria, resulting in the previously mentioned increase of bacterial and fungal membrane permeability [159,170,171].

Susceptibility of the pathogen to the EO depends of various factors, such as the composition of the EO in terms of active compounds, concentration of the EO and solubility in the media [35,172]. Exposure time of the pathogen to the oil and the persistency of the EO’s effects (depending highly on EO’s volatility) in time also are variability factors considering the efficiency of an EO towards a pathogen [27,173,174]. Growth conditions of the pathogen, such as pH, temperature and dioxygen availability in the media are reported as influencing factors regarding EO’s efficiency on a specific pathogen. Thymol has shown greater efficiency in anaerobic conditions [35,175], susceptibility of bacteria in contact with different EO has been demonstrated to be higher coupled with a low pH, especially in food [35,170,176,177] while temperature effects on antimicrobial activity are still controversial [35]. Some authors reported an increased activity coupled with a higher temperature [178,179] whereas others have shown a better efficiency with a decreasing temperature [180,181,182]. 

Regarding EO phytotoxic effects, visible symptoms such as growth decrease, severe chlorosis or leaves’ burning [150,183], were previously related to several key features, notably [133,183,184,185,186]:

- Mitosis inhibition;
- Decrease of the cellular respiration;
- Ion leakage and membrane depolarisation;
- Waxy cuticular layer removal;
- Decrease of the chlorophyll content;
- Oxidative damages through reactive oxygen species’ production;
- Microtubule polymerisation.

However, no comprehensive study regarding the detailed herbicidal mechanisms was previously published [183]. The same authors investigated cinnamon and Java citronella EO and some of their respective main chemical components, namely trans-cinnamaldehyde, citronellal and citronellol, regarding their herbicidal effects. They came to the conclusion that all above-mentioned EO or compounds were efficient herbicides against *A. thaliana,* most likely affecting the plant plasma membrane but not resulting in ion leakage. An effect on membrane domains and/or related properties was then put forward [183].

### 1.3. Market and Regulation

In the recent years, more and more studies about EO have been published, aiming to investigate their biological properties. Unfortunately, from the current perspective, EO are suffering a loss of efficiency when used as such in the field, owing mostly to their volatile nature and degradation susceptibility. In addition, their approval and registration procedure are very costly, because of the inherent cost of toxicity and environmental suitability assessments [9,187,188]. On a worldwide scale, biopesticides including biocontrol agents and EO are evaluated (as part of approval procedure) through the exact same procedure as their synthetic counterparts and similarly for the registration procedure [12,189,190]. A discussion is however currently initiated towards a relief of the process with a streamlined registration procedure for low-risk products (*i.e*., products that must not exert toxicity towards non target organisms and have a low persistency in soil [12]).

At the present time, biocontrol market (world-widely valued at approximately $3 billion) accounts for only 5% of the global crop protection sector but this is a relatively fast-growing market segment expecting to reach more than 7% of the total crop protection market by 2025 (more than $4.5 billion estimated) with an annual 8.84% growth estimation [191,192,193].

In comparison to the biocontrol products’ market, the global market for EO (natural cosmetics, beauty products, medicines and nutraceuticals) was estimated at about 4.9 billion euros in 2014, growing to 5.5 billion the next year and expected to reach more than 10 billion euros in 2020 [194]. Europe represents the biggest market, with a global market share of 40% in 2014 [194,195]. France is the second provider concerning high value EO to Europe, after the United States, though supplying relatively small [194]. In comparison, the global cosmetics market in the United States, Europe, China and Japan was worth 168 billion euros in 2014 [194].

The number of registered biocontrol agents and in our case more specifically EO-based products are heavily lower in Europe compared to the United States [193]. In the United States, the development of pesticides based on natural products has been facilitated, by exemption from registration for certain oils commonly used in processed foods and beverages [7,196,197]. Owing to this opportunity, some EO-based products have been developed and tested as fungicides, herbicides or insecticides, using thyme, clove and rosemary EO as active ingredients [7,196]. In particular, two American companies have commercialised several EO-based products, including Cinnamite and Valero from Mycotech Corporation (respectively an aphicide/miticide/fungicide and a fungicide) and EcoPCO, EcoTrol Plus, SPoran and Matran from EcoSMART Technologies (respectively insecticide, insecticide/miticide, fungicide and herbicide), among other products, namely Buzz Away or Green Ban [197,198].

In the recent years across Europe, however, an increasing number of EO has been homologated for use in agriculture, especially as biocides. Thus, EO from various plants and origins have been registered for specified uses (in particular biocidal effects), such as *Mentha arvensis* and *Mentha spicata*, *Juniperus mexicana*, *Citrus x sinensis*, *Persicaria odorata*, *Piper nigrum*, *Canarium commune*, *Cinnamomum zeylanicum*, *Boswellia carterii*, *Cymbopogon flexuosus*, *Litsea cubeba*, *Artemisia alba*, *Cistus ladaniferus*, *Copaifera tree*, *Ferula galbaniflua*, *Citrus aurantium* and *Schinus terebinthifolius* [199]. Commercial products are available for use in certain European countries, notably BIOXEDA (clove EO as a fungicide or bactericide on apple and pear trees storage pathogens), BIOX-M (Mentha spicata EO as a growth regulator on potato) or LIMOCIDE-OROCIDE-PREV-AM and ESSEN’CIEL (Sweet orange EO against whitefly, potato leafhopper, powdery mildew, blight, tobacco thrips on aromatic and medicinal plants, vegetable, fruit and ornamental crops as well as tobacco and vine). To this significant number of EO can be added extracts from aromatic plants (either main compounds or purified EO obtained by specific extraction technics such as supercritical fluid extraction), such as *Lavandula angustifolia*, *Artemisia alba*, *Citrus bergamia*, *Bulnesia sarmienti*, *Melaleuca leucadendron*, *Cinnamomum camphora*, *Elettaria cardamomum*, *Coriandrum sativum*, *Cupressus sempervirens*, *Eucalyptus globulus* and *Citrus paradisi* (non-exhaustive list [199]).

### 1.4. Innovative Avenue—Essential Oil Formulation

So as to legitimate and encourage EO application in agriculture as “green pesticides” and especially in the context of agroecology, it is necessary to find suitable options to promote their use, efficiency and persistence of effects in time. In particular, the stability of the EO when they are used in fields and the persistency of their effects in time are often brought forward as limited. In addition, working with EO might represent an expensive option because of the relatively low yield of obtention and a costly approval procedure. A recent study on rosemary EO [130] has put forth encapsulation (starch coating) of the EO as a means to slowly release it in the soil and control the EO diffusion as well as to improve its efficiency as a potential bio-herbicide with a perspective of field use.

From a wider perspective, a product formulation is a homogeneous and stable mixture of active and inert ingredients [200], involving a specific processing of the product to enhance its biological properties as well as their durability and the stability of the product. Because many of them are not suitable for use in their raw state (toxicity, poor solubility, instability, etc., [200,201]), formulations are commonly used for pesticides and the same kind of technic should be applicable to EO so as to obtain similar benefits. Additionally, coating materials are bio-sourced and biodegradable products [202]. One should note that even though these two technics may represent promising avenues, the choice of a formulation highly depends on the intended use and application method, the target pathogen as well as the potential environmental degradation factors [201,203].

#### 1.4.1. Essential Oils Emulsification

First, an emulsion is a mixture of two immiscible and suspended phases in a liquid state, often in the presence of a surfactant which acts as a stabiliser [200]. Based on this consideration, emulsion may find use for the enhancement of EO’s stability.

Emulsions are primarily classified according to their particle diameter and their thermodynamic stability as macroemulsions, nanoemulsions or microemulsions. The nanoemulsion formulations of active substances, including EO, can be used to develop biodegradable coating to enhance both their quality and biological properties [204].

The preparation methods can be distinguished between high-energy (high-pressure or mechanical homogenisation and ultra-sonication) and low energy (divided into isothermal and thermal processes [205]) methods [204]. One should notice that the choice of both surfactant and appropriate formulation are key components in an efficient pathogen management [203].

Specifically, macroemulsions have a wider particle size and are susceptible to break down overtime because of destabilising factors (gravitational separation, flocculation, coalescence, Ostwald ripening [204,205]) and appear turbid because of the particle size which is similar to the light’s wavelength. When it comes to nanoemulsions, they are metastable system as well, but the smaller droplet’s size confers a better stability regarding gravitational and aggregation phenomena, which would be beneficial regarding EO stability. Regarding microemulsions, they share similar droplets’ size with nanoemulsions, but they are thermodynamically stable [200,204,206]. By using nanoemulsions in particular, EO stability issues could be solved and the fine droplet size would also be beneficial by increasing cellular absorption and hence enhancing EO’s biological properties [207]. Additionally, both nano- and microemulsions are o/w emulsions, consisting of three main components: oil phase dissolved in organic solvent, water and surfactants and cosurfactants in varying amounts [200]. Eventually, because of their nature and the preparation process, nanoemulsions require smaller amounts of surfactant and are of greater economic interest [200,204,205].

In brief, EO emulsions and in particular nanoemulsions could be of great interest in ensuring either a controlled release of the EO compounds and a better stability of the product or enhanced biological properties [203]. Several studies have already demonstrated higher efficiency [208,209,210,211,212], stability or even enhanced bioavailability [213,214] when EO were prepared in nanoemulsions.

#### 1.4.2. Essential Oils Encapsulation

Encapsulation of the EO is another emerging technic with the potential to enhance EO stability and provide a controlled-release of the product [27,215,216].

There are different processes leading to the obtention of either micro- or nano-capsules. The basic concept of EO encapsulation is summarised as the process of surrounding a particle or molecule of interest with a coating or building a functional barrier between a core and wall material, so as to avoid physical and chemical reactions between the core and the outer molecules [175]. This technic aims in maintaining the properties of the core material (for example EO) such as biological, functional and physicochemical properties and avoid deterioration [27,175,216]. Two processes are mostly used: coacervation and spray-drying. The spray-drying process is a common encapsulation technic used on an industrial scale, which presents the advantages of producing microcapsules in a relatively simple process and that while being quite inexpensive in comparison with other encapsulation technics [175]. This process requires the use of an emulsion. It relies on the atomisation of EO emulsions into a drying chamber, at a relatively high temperature, leading to a very fast water evaporation and therefore a quasi-instantaneous entrapment of the EO in a fast-formed crust [175,217]. The coacervation process is another widely used encapsulation technic. This technic relies on a phase separation: it involves an electrostatic attraction between biopolymers, which leads to a separation of a liquid phase from the polymer-rich phase, also called coacervate [175]. Coacervation is distinguished into two different processes, either simple or complex [175,218,219,220].

The encapsulation of EO in polymeric particles has been investigated: in oligomer particles such as cyclodextrins [221,222], in biopolymers [223,224] or in microparticles or microcapsules through complex coacervation [225,226,227]. Lavandin EO, successfully encapsulated in a biodegradable polymer, displayed a narrow particle size and an appropriate time-release curve favourable for a controlled release of the product [224]. Similarly, *Mentha x piperita* EO in the presence of cyclodextrins was found forming guest-host complexes, hence allowing to obtain a controlled release [38,221].

There is however a consequent downside during the process, as it is required to heat or evaporate during the encapsulation, which is risky when it comes to EO [27]. Encapsulation in liposomes, which are amphiphilic molecules able to ‘self-organise’ in layers in aqueous media, defining several aqueous compartments, is also feasible. These liposomes are commonly used as carriers for other molecules (either hydrophilic, lipophilic or even amphiphilic) in different compartments [228]. The encapsulation in solid lipid nanoparticles (SLN), which can be constituted by lipid or lipid-like molecules, such as waxes or triacylglycerols [27,229,230], is another advanced technology providing the same advantages as previously mentioned [231]. *Artemisia arborescens* EO encapsulated in SLN was demonstrated significantly more stable in comparison with the raw EO [229].

In brief, encapsulation would represent an innovative method to enhance EO stability and efficacy, by solving some of the downsides EO present when used raw. In particular, the issue regarding a reduction of the EO effects when applied in fields may be solved by means of controlled release through the EO encapsulation. The potential of nanotechnologies regarding EO encapsulation has already been reviewed [232]. Moreover, a few studies link above-cited properties with a potential use in agriculture, for either EO [223,224,229,233] or secondary metabolites in a broader sense [234].

## 2. Conclusions

In the recent years, a large number of studies have been focusing on EO as a source for new biopesticides. A relative effectiveness against pathogens, multiple mechanisms of action and a relatively low toxicity to mammals and human beings have in particular been highlighted [151,235]. However, a small number of them have been homologated and is permitted for use worldwide. This number of EO homologated in agriculture for various biocide usages (herbicide, fungicide and insecticide) as well as being usable as growth regulator, is remaining surprisingly scarce. This could be explained by the different constraints that EO are facing.

Tests including EO properties are in most cases a screening of the properties against one or several pathogens with a narrow screening spectrum [236] and more importantly tests are commonly run *in vitro*, only a few include a glasshouse in planta experiment at least and even scarcer is the number of *in situ* experiments. It is well-known that EO are facing an efficiency drop when used raw in the fields, mostly owing to their volatility. Yet, the fact that the interaction plant-pathogen-EO is not well studied or published leads to a lack of knowledge when it comes to the field, worsening the efficiency drop while not encouraging the use of EO-based products in biocontrol [196].

Because of their stability issues, EO may in addition be affected during either their storage or transport [7]. It should then be kept in mind that although the contact effect of some EO against pathogens is good, a fast volatilisation when used in agriculture could lead to a low persistence of the effect in time, giving more credit to the importance of EO formulation [235]. But once again, field applications’ reports remain scarce, which might be due to the recent emergence of the nano-emulsion technology that only appeared in the middle of the nineties [237] or to a possible lack of efficiency in the current state. Research should be able to provide insights towards the commercialisation of EO-based products at least as efficient as in controlled laboratory conditions, with an improved stability of the compounds under field conditions, yet there is still a lack of knowledge when it comes to the field. On a societal aspect, it appears necessary to work on essential points so as to encourage EO’s use and avoid understanding issues regarding in particular negative and false public perception (e.g., higher cost of the final product for the consumers, health risks due to the use of EO) as well as farmers concerns about the effectiveness of the products [8,190]. As an example, providing a risk assessment of EO’s effects on non-target organisms would be beneficial, as well as demonstrating, under field conditions, the efficiency of EO, free or formulated.

Finally, the authorisations regarding biopesticides commercialisation (and synthetic pesticides even more) are granted through a very complex and onerous process [9,238]. These require in particular evidence from a certain number of tests, such as toxicological and environmental studies or efficiency. In many cases, studies on toxicological effects do not exist and are too expensive and time-consuming for local manufacturers for example [198,235]. One of the big constraint in commercialising EO products as biopesticides could then be a consequence of regulatory barriers [196]. Nonetheless, the current trend towards a reduction of synthetic pesticides’ use as well as an alleviation of the approval procedure for low-risk substances might enable EO products to be developed and used worldwide.

One should keep in mind that biocontrol is not without its own risks as well [239]. In the present case with EO, the environmental risk is significantly lower, owing to the volatile nature of EO, which leads to a significant reduction in terms of persistency in comparison with synthetic pesticides. Non-target living organisms in the ecosystem may be less impacted, due to a minor residual activity of the EO and EO-based products used as biopesticides [196]. Yet, even though the aim of EO formulation is to provide enhanced stability and efficiency of EO and that coating materials are most the time not toxic for living organisms themselves, nanotechnologies could face undesirable effects, in particular because of the interaction with the highly reactive surface of several types of nanomaterials (especially those containing metallic nanoparticles [202,234]). A careful evaluation regarding their potent toxicity should then be carried out [234].

New emerging techniques such as EO formulation through emulsion or encapsulation might enable EO to appear on a wider scale, as a means to enhance both their biological activity and stability, with considerations however from an economic point of view. In that concern, both technics might represent innovative methods for EO to emerge on the market as viable biocontrol products. A feasible and gentle transition could be the use of EO, preferentially formulated according to the previous considerations, in a traditional pesticide crop management system, in complement with synthetic pesticides, allowing to reduce the amounts of pesticides used towards an integrated pest management system. 

## Figures and Tables

**Table 1:** Antifungal properties of essential oils (EO) against phytopathogenic fungi and oomycetes studied during the last decade.

Target Organism		Disease Caused by the Pathogen	Essential Oil Distilled from	References
***Fungi***	*Alternaria alternata*	Leaf spot, alternariose	*Carum carvi* L., *Carum opticum* L. and *Foeniculum vulgare* L.	[58]
			18 egyptian plants	[59]
			*Echinophora platyloba* (seed)	[60]
			*Thuja plicata*, *Eugenia caryophillata* L., *Lavandula angustifolia*, *Origanum vulgare* L., *Salvia sclarea* and *Thymus vulgaris* L.	[61]
			*Thymus zygiis*	[62]
			*Laurus nobilis*	[63]
	*Alternaria humicola*	Alternariose	*Asarum heterotropoides*	[64]
	*Alternaria solani*	Early blight	*Angelica archangelica*	[65]
	*Alternaria spp.*	Alternariose	*Pinus pinea*	[25]
			*Genista quadriflora*	[66]
			*Pulicaria mauritanica*	[67]
			*Warionia saharae*	[68]
	*Aspergillus carbonarius*	Ochratoxin producer	*Citrus x limon* L.	[69]
	*Aspergillus flavus*	Rot and mould, aflatoxins production, aspergillosis	*Mentha x piperita*, *Origanum* spp., *Rosmarinus officinalis* L., *Schinus mole* L. and *Tagetes minuta* L.	[70]
			*Eucalyptus* sp., *Ferula galbaniflua*, *Thymus capitatus* and *Syzygium aromaticum*	[71]
			*Curcuma longa*	[72]
			*Angelica glauca, Plectranthus rugosus, Valeriana wallichii*	[73]
			*Mentha spicata*	[74]
			*Michelia alba*	[75]
			*Ocimum basilicum* and *Vetiveria zizanioides*	[76]
			*Artemisia nilagirica*	[77]
	*Aspergillus fumigatus*	Rot and mould, aflatoxins production, aspergillosis	*Santolina chamaecyparissus*	[78]
			*Thuja plicata*, *Eugenia caryophillata* L., *Lavandula angustifolia*, *Origanum vulgare* L., *Salvia sclarea* and *Thymus vulgaris* L.	[61]
	*Aspergillus niger*	Mould	*Ocimum basilicum* L.	[79]
			*Genista quadriflora*	[66]
			*Ocimum basilicum* L.	[80]
			*Lallemantia royleana*	[81]
			*Artemisia nilagirica*	[77]
			*Ocimum basilicum* and *Vetiveria zizanioides*	[76]
			*Solidago canadensis* L.	[82]
			*Marrubium vulgare*	[83]
	*Aspergillus ochraceus*	Ochratoxin producer	*Artemisia nilagirica*	[77]
	*Aspergillus parasiticus*	Mould	*Citrus x limon* L.	[69]
	*Aspergillus spp.*		*Cinnamomum zeylanicum*, *Thymus vulgaris* L., *Origanum vulgare* L., *Syzygium aromaticum* L.), *Cymbopogon citratus* and *Zingiber officinale* Rosc.	[51]
			14 different botanical plant species	[72]
	*Bipolaris oryzae*	Brown spot	*Piper sarmentosum*	[73]
***Fungi***	*Bipolaris sorokiniana*	Leaf blight/spot	*Pinus pinea*	[25]
			*Eucalyptus erythrocorys*	[84]
	*Biscogniauxia mediterranea*	Charcoal disease	*Eucalyptus* spp.	[54]
	*Botryotinia fuckeliana*	Grey mould	*Thymus zygiis*	[62]
	*Botrytis cinerea*	Grey mould	*Cestrum nocturnum*	[85]
			*Carum carvi* L., *Foeniculum vulgare* L. and *Mentha* x *piperita*	[50]
			18 egyptian plants	[59]
			*Mentha pulegium*	[86]
			*Metasequoia glyptostroboides*	[87]
			*Origanum heracleoticum*	[88]
			*Origanum majorana*	
			*Eucalyptus erythrocorys*	[84]
			*Tetraclinis articulata*	[89]
			*Thymus* spp.	[90]
			*Melissa officinalis*	[91]
			*Cinnamomum cassia*	[92]
			*Angelica archangelica*	[65]
			*Solidago canadensis* L.	[82]
			*Melaleuca alternifolia*	[93]
			*Tetraclinis articulata*	[94]
			*Marrubium vulgare*	[83]
	*Choanephora cucurbitarum*	Fruit and blossom rot	*Cinnamomum camphora*	[95]
			*Syzygium cumini*	[96]
	*Cladosporium cladosporioides*	Rot	*Citrus x limon* L.	[69]
			*Thuja plicata*, *Eugenia caryophillata* L., *Lavandula angustifolia*, *Origanum vulgare* L., *Salvia sclarea* and *Thymus vulgaris* L.	[61]
	*Colletotrichum capsici*	Leaf spot	*Cestrum nocturnum*	[85]
			*Metasequoia glyptostroboides*	[87]
			*Piper chaba*	[97]
	*Colletotrichum gloeosporioides*	Leaf spot	*Cymbopogon* sp.	[98]
			*Asarum heterotropoides*	[64]
	*Colletotrichum tricbellum*	Leaf spot	*Echinophora platyloba* (seed)	[60]
	*Curvularia fallax*	Black sheath spot—Leaf spot		
	*Cytospara sacchari*	Stem canker on sugarcane		
	*Eurotium herbariorum*	Mould	*Citrus x limon* L.	[69]
	*Fusarium avenaceum*	Ear blight and root rot of cereals	*Eucalyptus erythrocorys*	[84]
	*Fusarium oxysporum*	Fusarium wilt (vascular disease)	18 egyptian plant species	[59]
			*Metasequoia glyptostroboides*	[87]
			*Eucalyptus erythrocorys*	[84]
***Fungi***	*Fusarium oxysporum*	Fusarium wilt (vascular disease)	*Genista quadriflora*	[66]
			*Echinophora platyloba* (seed)	[60]
			*Piper chaba*	[97]
			*Syzygium aromaticum*, *Eucalyptus globulus*, *Cymbopogon citratus* and *Mentha x piperita*	[56]
			*Mikania scandens*	[57]
			*Salmea scandens*	[99]
	*Phytophthora megakarya*	Black pod disease	*Syzygium aromaticum* and *Zanthoxylum xanthoxyloides*	[55]
	*Pythium* spp.	Root rot	*Thymus* spp.	[90]
			*Mikania scandens*	[57]
	*Fusarium solani*	Root rot, soft rot of plant tissues	18 egyptian plant species	[59]
			*Metasequoia glyptostroboides*	[87]
			*Eucalyptus erythrocorys*	[84]
			*Asarum heterotropoides*	[64]
			*Angelica glauca, Plectranthus rugosus, Valeriana wallichii*	[73]
			*Piper chaba*	[97]
			*Marrubium vulgare*	[83]
	*Fusarium spp.*		*Cestrum nocturnum*	[85]
			*Pinus pinea*	[25]
			*Rosmarinus officinalis*	[100]
			*Tetraclinis articulata*	[90]
			*Angelica archangelica*	[65]
			14 different botanical plant species	[101]
	*Fusarium sulphureum*	Dry rot	*Zanthoxylum bungeanum*	[102]
	*Fusarium verticillioides*	Ear rot on maize	*Curcuma longa*	[103]
	*Geotrichum citri-aurantii*	Sour rot (post-harvest)	*Thymus* spp.	[104]
	*Lasiodiplodia theobromae*	Rot and dieback (forest species)	*Myrcia lundiana*	[105]
	*Macrophomina phaseolina*	Damping-off, seedling blight, rot	*Mentha x piperita* and *Ocimum basilicum*	[106]
			*Echinophora platyloba* (seed)	[60]
	*Microdochium nivale*	Patch lawn disease	*Pinus pinea*	[25]
	*Monilinia fructicola*	Brown rot	*Mentha pulegium*	[86]
			*Solidago canadensis* L.	[82]
	*Penicillium digitatum*	Green mould (post-harvest)	*Carum carvi* L., *Carum opticum* L. and *Foeniculum vulgare* L.	[58]
			*Foeniculum vulgare* Mill., *Satureja hortensis* L., *Ocimum basilicum* L. and *Thymus vulgaris* L.	[53]
			*Thymus* spp.	[104]
			*Marrubium vulgare*	[83]
	*Penicillium expansum*	Post-harvest mould	*Melissa officinalis*	[91]
			*Pulicaria mauritanica*	[67]
			*Solidago canadensis* L.	[82]
			*Warionia saharae*	[68]
***Fungi***	*Penicillium italicum*	Blue mould	*Thymus* spp.	[104]
			*Rosmarinus officinalis*	[100]
	*Penicillium* spp.		*Mentha x piperita*, *Origanum* spp., *Rosmarinus officinalis* L., *Schinus mole* L. and *Tagetes minuta* L.	[70]
			*Citrus x limon* L.	[69]
			*Ocimum basilicum*	[79]
			*Ocimum basilicum*	[80]
			*Ocimum basilicum* and *Vetiveria zizanioides*	[76]
			14 different botanical plant species	[101]
	*Penicillium verrucosum*	Ochratoxin producer	Residues of *Lamiceae* species	[52]
			*Allium sativum* L., *Mentha x piperita, Origanum onites* L. and *Salvia officinalis* L.	[107]
	*Rhizoctonia solani*	Damping-off, root and stems rot	*Cestrum nocturnum*	[85]
			*Metasequoia glyptostroboides*	[87]
			*Asarum heterotropoides*	[64]
			*Angelica archangelica*	[65]
			*Bunium persicum, Foeniculum vulgare, Juniperus polycarpus, Mentha* spp., *Ocimum basilicum, Thymus vulgaris* and *Zingiber officinale*	[106]
			*Piper chaba*	[97]
			*Syzygium cumini*	[96]
			*Thymus* spp.	[90]
			*Mikania scandens*	[57]
			*Piper sarmentosum*	[108]
	*Rhizoctonia sp.*		*Pinus pinea*	[25]
	*Rhizopus microsporus*	Rice seedling blight, various head, grain and ear rots	*Ocimum basilicum* and *Vetiveria zizanioides*	[76]
	*Rhizopus stolonifer*	Storage/post-harvest rot	*Foeniculum vulgare* Mill., *Satureja hortensis* L., *Ocimum basilicum* L. and *Thymus vulgaris* L.	[53]
			*Melissa officinalis*	[91]
			*Pulicaria mauritanica*	[67]
			*Warionia saharae*	[68]
	*Sclerotinia sclerotiorum*	White mould	*Cestrum nocturnum*	[85]
			*Metasequoia glyptostroboides*	[87]
			*Ziziphora clinopodioides*	[109]
	*Verticillium dahliae*	Verticillium wilt	35 plant’s botanical species	[110]
	*Villosiclava virens*	Rice false smut	18 plant’s botanical species	[111]
***Oomycetes***	*Phytophthora cactorum*	Root rot	*Asarum heterotropoides*	[64]
	*Phytophthora capsici*	Blight	*Cestrum nocturnum*	[85]
			*Metasequoia glyptostroboides*	[87]
			*Piper chaba*	[97]
	*Phytophthora infestans*	Late blight	*Salmea scandens*	[99]
			*Citrus sinensis* Cadenera, *Citrus limon* Eureka and *Citrus bergamia* Castagnaro	[112]
			*Thymus* spp.	[90]
			*Origanum majorana* L.	[113]
***Oomycetes***	*Phytophthora megakarya*	Black pod disease	*Syzygium aromaticum* and *Zanthoxylum xanthoxyloides*	[55]
	*Pythium* spp.	Root rot	*Thymus* spp.	[90]
			*Mikania scandens*	[57]

**Table 2:** Examples of EO acting against phytopathogenic bacteria (from 2007 to present).

Essential Oil Distilled from	Target Bacteria and Caused Disease			References
*Achillea biebersteinii*	**Gram positive bacteria**	*Clavibacter michiganensis*	Ring rot disease	[119]
*Achillea millefolium*				
*Ocimum ciliatum*		*Rhodococcus fascians*	Leafy gall disease	[116]
*Origanum heracleoticum*		*Clavibacter michiganensis*	Ring rot disease	[88]
*Origanum majorana*				
*Origanum onites*				[116]
*Salmea scandens*				[99]
*Satureja hortensis*				[120]
*Satureja spicigera*				[119]
*Solidago canadensis* L.				[82]
*Tanacetum aucheranum*				[117]
*Thymus fallax*				[119]
*Achillea biebersteinii*	**Gram negative bacteria**	*Erwinia spp.*		[119]
		*Pseudomonas spp.*	Bacterial canker	
		*Xanthomonas spp.*	Bacterial spots and blights	
*Achillea millefolium*		*Erwinia spp.*		
		*Pseudomonas spp.*	Bacterial canker	
		*Xanthomonas spp.*	Bacterial spots and blights	
*Citrus aurantium* L.		*Agrobacterium tumefaciens*	Crown gall	[121]
		*Dickeya solani*	Black leg and soft rot	
		*Erwinia amylovora*	Fire blight	
*Citrus reticulata*		*Pseudomonas aeruginosa*	Soft rot	[122]
*Cleistocalyx operculatus*		*Xanthomonas spp.*	Bacterial spots and blights	[123]
*Cynara scolymus* (stems)	**Gram negative bacteria**	*Erwinia amylovora*	Fire blight	[124]
		*Erwinia carotovora*	Soft rot	
		*Pseudomonas syringae*	Bacterial canker	
		*Xanthomonas vesicatoria*	Bacterial leaf spot	
*Eriocephalus africanus* L.		*Agrobacterium tumefaciens*	Crown gall	[125]
		*Dickeya solani*	Black leg and soft rot	
		*Erwinia amylovora*	Fire blight	
		*Pseudomonas cichorii*	Leaf blight and spots	
		*Serratia pulmithica*		
*Juglans regia* L. (shells)		*Erwinia amylovora*	Fire blight	[124]
		*Erwinia carotovora*	Soft rot	
		*Pseudomonas syringae*	Bacterial canker	
		*Xanthomonas vesicatoria*	Bacterial leaf spot	
*Metasequoia glyptostroboides*		*Xanthomonas spp.*	Bacterial spots and blights	[89]
*Ocimum ciliatum*		*Agrobacterium vitis*	Crown gall	[116]
		*Brenneria nigrifluens*	Cankers	
		*Pantoea stewartii*	Stewart’s wilt and leaf blight	
		*Pseudomonas spp.*	Bacterial canker	
		*Ralstonia solanacearum*	Bacterial wilt	
		*Xanthomonas spp.*	Bacterial spots and blights	[123]
*Ocimum basilicum*		*Pseudomonas aeruginosa*	Soft rot	[76]
*Origanum heracleoticum*		*Pseudomonas* spp.	Bacterial canker	[88]
		*Xanthomonas* sp.	Bacterial spots and blights	
*Origanum majorana*		*Pseudomonas* spp.	Bacterial canker	
		*Xanthomonas* sp.	Bacterial spots and blights	
*Origanum onites*		*Erwinia spp.*		[116]
		*Pseudomonas spp.*	Bacterial canker	
		*Xanthomonas spp.*	Bacterial spots and blights	
*Origanum vulgare*		*Erwinia amylovora*	Fire blight	[124]
		*Erwinia carotovora*	Soft rot	
		*Pseudomonas syringae*	Bacterial canker	
		*Xanthomonas vesicatoria*	Bacterial leaf spot	
		*Pseudomonas syringae*	Bacterial canker	[126]
		*Pseudomonas spp.*	Bacterial canker	[127]
*Piper sarmentosum*		*Xanthomonas oryzae pv. oryzae*	Bacterial blight	[108]
		*Xanthomonas oryzae pv. oryzicola*	Bacterial blight	
*Salmea scandens*		*Pseudomonas syringae*	Bacterial canker	[99]
		*Erwinia carotovora*	Soft rot	
		*Erwinia spp.*		
		*Pseudomonas spp.*	Bacterial canker	
*Salmea scandens*		*Xanthomonas spp.*	Bacterial spots and blights	[99]
		*Erwinia carotovora*	Soft rot	
*Satureja hortensis*		*Erwinia spp.*		[120]
		*Pseudomonas spp.*	Bacterial spots and blights	
		*Xanthomonas spp.*	Bacterial spots and blights	
*Satureja spicigera*		*Erwinia spp.*		[118]
		*Pseudomonas spp.*	Bacterial canker	
		*Xanthomonas spp.*	Bacterial spots and blights	
*Solidago canadensis* L.		*Pseudomonas* spp.	Bacterial canker	[82]
		*Xanthomonas* sp.	Bacterial spots and blights	
*Syzygium aromaticum*	**Gram negative bacteria**	*Erwinia amylovora*	Fire blight	[124]
		*Erwinia carotovora*	Soft rot	
		*Pseudomonas syringae*	Bacterial canker	
		*Xanthomonas vesicatoria*	Bacterial leaf spot	
*Tanacetum aucheranum*		*Agrobacterium tumefaciens*	Crown gall	[117]
		*Erwinia spp.*		
		*Pseudomonas spp.*	Bacterial canker	
		*Xanthomonas spp.*	Bacterial spots and blights	
*Tanacetum chiliophyllum*		*Agrobacterium tumefaciens*	Crown gall	[118]
		*Erwinia spp.*		
		*Pseudomonas spp.*	Bacterial canker	
		*Xanthomonas spp.*	Bacterial spots and blights	
*Thymus fallax*		*Erwinia spp.*		[126]
		*Pseudomonas spp.*	Bacterial canker	
		*Xanthomonas spp.*	Bacterial spots and blights	
*Thymus vulgaris*		*Pseudomonas syringae*	Bacterial canker	[127]
		*Pseudomonas spp.*	Bacterial canker	
*Vetiveria zizanioides*		*Pseudomonas aeruginosa*	Soft rot	[76]
*Zataria multiflora*		*Xanthomonas campestris*	Black rot and leaf spot	[128]
11 different plants		*Erwinia amylovora*	Fire blight	[129]
18 egyptian plants		*Agrobacterium tumefaciens*	Crown gall	[59]
		*Erwinia carotovora*	Soft rot	

**Table 3:** Examples of EO presenting herbicidal properties.

Essential Oil Distilled from	Plant Tested	References
*Achillea gypsicola*	*Amaranthus retroflexus*	[137]
	*Chenopodium album*	
	*Cirsium arvense*	
	*Lactuca serriola*	
	*Rumex crispus*	
*Achillea biebersteinii*	*Amaranthus retroflexus*	[137]
	*Chenopodium album*	
	*Cirsium arvense*	
	*Lactuca serriola*	
	*Rumex crispus*	
*Angelica glauca*	*Lemna minor*	[73]
*Citrus x limon* L.	*Portulaca oleracea*	[140]
*Citrus aurantiifolia*	*Avena fatua*	[131]
	*Echinochloa crus-galli*	
	*Phalaris minor*	
*Coriandrum sativum* L.	*Amaranthus retroflexus*	[141]
	*Chenopodium album*	
	*Echinochloa crus-galli*	
*Eucalyptus* spp.	*Annual ryegrass*	[142]
	*Echinochloa crus-galli*	[143]
	*Lolium multiflorum*	
	*Nicotiana glauca*	
	*Phalaris minor*	[139]
	*Parthenium hysterophorus*	[138]
	*Portulaca oleracea*	[143]
	*Sinapis arvensis*	[84]
	*Phalaris canariensis*	[36]
	*Solanum elaeagnifolium*	
*Lavandula* spp.	*Lolium rigidum*	[144]
12 Mediterranean species	*Lactuca sativa*	[136]
	*Lepidium sativum*	
	*Raphanus sativus*	
*Origanum acutidens*	*Amaranthus retroflexus*	[135]
	*Rumex crispus*	
	*Chenopodium album*	
*Origanum vulgare* L.	*Hordeum vulgare*	[145]
	*Lepidium sativum*	
	*Matricaria chamomilla* L.	[146]
	*Sinapsis alba*	[145]
	*Triticum aestivum*	
*Peumus boldus*	*Portulaca oleracea*	[140]
*Pinus nigra*	*Phalaris canariensis*	[147]
	*Trifolium campestre*	
	*Sinapis arvensis*	
*Pinus pinea*	*Sinapis arvensis*	[25]
	*Raphanus raphanistrum*	
	*Lolium rigidum*	
*Plectranthus rugosus*	*Lemna minor*	[73]
*Rosmarinus officinalis*	*Amaranthus retroflexus*	[130]
	*Matricaria chamomilla* L.	[146]
	*Phalaris minor*	[100]
	*Rhaphanus sativus*	[130]
	*Silybum marianum*	[100]
	*Trifolium incarnatum*	
*S* *yzygium aromaticum*	Common lambsquarters	[148]
	*Redwood pigweed*	
*Tagetes erecta*	*Echinochloa crus-galli* L. Beauv.	[149]
*Tanacetum aucheranum*	*Amaranthus retroflexus*	[117]
	*Chenopodium album*	
	*Rumex crispus*	
*Tanacetum chiliophyllum*	*Amaranthus retroflexus*	
	*Chenopodium album*	
	*Rumex crispus*	
*Tetraclinis articulata*	*Sinapis arvensis*	[89]
	*Phalaris canariensis*	
25 various plants	*Taraxacum officinale*	[150]
*Valeriana wallichii*	*Lemna minor*	[73]
